# Coupled Electromagnetic-Dynamic Modeling and Bearing Fault Characteristics of Induction Motors considering Unbalanced Magnetic Pull

**DOI:** 10.3390/e24101386

**Published:** 2022-09-28

**Authors:** Liangyuan Huang, Guoji Shen, Niaoqing Hu, Ling Chen, Yi Yang

**Affiliations:** 1 College of Intelligence Science and Technology, National University of Defense Technology, Changsha 410073, China; 2 Laboratory of Science and Technology on Integrated Logistics Support, NUDT, Changsha 410073, China

**Keywords:** induction motor, bearing faults, coupled model, unbalanced magnetic pull, multiple coupled circuit

## Abstract

Induction motors are complex energy conversion systems across the domains of dynamics, electricity, and magnetism. Most existing models mainly consider unidirectional coupling, such as the effect of dynamics on electromagnetic properties, or the effect of unbalanced magnetic pull on dynamics, while in practice it should be a bidirectional coupling effect. The bidirectionally coupled electromagnetic-dynamics model is beneficial to the analysis of induction motor fault mechanisms and characteristics. This paper proposes a coupled electromagnetic-dynamic modeling method that introduces unbalanced magnetic pull. By using the rotor velocity, air gap length, and unbalanced magnetic pull as the coupling parameters, the coupled simulation of the dynamic and electromagnetic models can be effectively realized. Simulation results for bearing faults show that the introduction of magnetic pull induces a more complex dynamic behavior of the rotor, which in turn leads to modulation in the vibration spectrum. The fault characteristics can be found in the frequency domain of the vibration and current signals. Through the comparison between simulation and experimental results, the effectiveness of the coupled modeling approach and the frequency domain characteristics caused by the unbalanced magnetic pull are verified. The proposed model can help to obtain a variety of information that is difficult to measure in reality and can also serve as a technical basis for further research on nonlinear characteristics and chaos in induction motors.

## 1. Introduction

Induction motors are the most widely used electromechanical energy conversion devices in the industrial production chain, which benefits from the significant advantages of their simple construction in terms of easy maintenance and reliable operation. However, while most induction motors are inexpensive and easy to replace, their importance in the task chain may not match the cost of maintenance. In mission situations where unplanned interruptions are not tolerated, the cost of induction motor faults can be incalculable. According to the statistics of IEEE-IAS and EPRI, for a large number of small and medium-sized motor faults, more than 40% of induction motor faults are related to bearings [1,2,3,4]. Therefore, the research on mechanism and prevention of induction motor bearing faults has been widely concerned.

Vibration from motor bearings can be transmitted to motor end shields and enclosures; thus, it seems that many of the vibration analysis methods applied to common bearings can be easily transferred to motors. Based on vibration signals, many research results on bearing faults have been published over the years, including, but not limited to, modeling and simulation, signal processing, feature extraction, life prediction, and intelligent diagnosis. Taking some of the recently published studies as examples, Zhao et al. [5] proposed a performance degradation prediction method based on high-order differential mathematical morphology gradient spectrum entropy, phase space reconstruction, and extreme learning machines to predict the performance degradation trend of rolling bearings. Zhu et al. [6] used a hidden Markov model to locate the fault occurrence time and solved the distribution discrepancy problem by a transfer learning method based on multiple layer perceptron. Cui et al. [7] proposed a coupled multistable stochastic resonance method with two first-order multistable stochastic resonance systems, which has higher output signal-to-noise ratio than multistable stochastic resonance. Dong et al. [8] used a bearing dynamics model to generate a large amount of simulated data for the small sample problem, and then implemented transfer learning between the diagnostic knowledge in the simulated data to the real scenario based on convolutional neural network and parameter transfer strategies. Considering the compensation balance excitation caused by the rotor mass eccentricity, Liu et al. [9] developed a dynamic model for the bearing rotor system of a high-speed train under variable speed conditions. The use of the angle iteration method in this case solves the problem that the space position of the rollers cannot be determined during bearing rotation. With the improvement of computer computing power and the rise of artificial intelligence algorithms, a large number of intelligent diagnostics works with excellent performance in public data sets have been published in recent years. However, how to explain the artificial intelligence model at the physical level and how to obtain enough fault sample data are still the key issues for the intelligent diagnosis method. By automatically generating data, the modeling approach that simulates machine faults makes it possible to solve the latter. Analytical models constructed according to the laws of physics can simulate faults of different sizes and locations and generate enough representative signals to train intelligent diagnostic algorithms. In addition, for complex cases occurring in the machine such as nonlinearity and coupling, an analytical model helps the understanding and analysis at the physical level [10].

Unlike general mechanical structures, induction motors are complex energy conversion systems with mechanism–electricity–magnetism coupling, which requires more detailed consideration in the research on it. However, as noted above, most studies focused on motor bearing faults have adopted analytical approaches similar to those of nonmotor bearing studies. Although they seem to work from the results, many simple transfer schemes ignore the electromagnetic force between the stator and rotor, i.e., the unbalanced magnetic pull (UMP) on the rotor. There have been studies and discussions about UMP since the beginning of the last century [11]; one of the reasons is to reduce the air gap in the design for improving the power factor [12]. The small air gap in rotating motors is susceptible to small variations in the dimensions of stator, rotor, bearings, etc. A nonuniform air gap will twist the flux density distribution, thus producing a UMP that tends to drag the rotor further away from its center position, which is difficult to ignore during motor operation. Therefore, UMP has been a long-standing concern in the electrical field, from early discussions of computational methods to current research on the dynamic behavior of motor rotors. Taking some of the research in the last three decades as an example, Dorrell and Smith [13,14] proposed a general analytical model for calculating the UMP in three-phase squirrel-cage induction motors with static eccentricity and observed a favorable correlation between the measured and calculated results. In another study, Dorrell [15] considered the axial variation of the eccentricity in the calculation for the UMP. Guo [16] et al. derived an analytical expression of the UMP caused by eccentricity in a three-phase motor at no load and investigated the nonlinear vibration response excited by the UMP in the Jeffcott rotor model. Using the same Jeffcott rotor model, Chen et al. [17] derived an analytical expression for the UMP in a permanent magnet synchronous motor and introduced it into the nonlinear vibration equation to investigate the variation of the system response under different design parameters. To investigate the rotor dynamics stability, Han et al. [18] proposed a method to calculate the radial and axial motor eccentric forces by magnetic equivalent circuit modeling. Zhang et al. [19] developed a rotor bearing system dynamics model considering gyroscopic effect, nonlinear bearing force, and UMP force, discovering the coupling effect between bearing force, unbalanced mass force, and UMP. From the published literature discussing UMP, many studies have focused on the effect of UMP on rotor dynamics.

On the other hand, studies on the dynamics of rotor-bearing systems have also led to many results. Cheng [20] et al. investigated the nonlinear dynamic behaviors of a rotor-bearing-seal coupled system by using the nonlinear seal fluid dynamic force model and nonlinear oil film force. Li et al. [21] proposed a generalized dynamics model for a rolling bearing-rotor system by coupling an explicit finite element model with a dynamic bearing model. Li et al. proposed a generalized dynamics model for a rolling bearing-rotor system by coupling an explicit finite element model with a dynamic bearing model. Yan et al. [22] developed a fractional order mathematical model of the rotor-bearing-seal system and investigated the dynamic characteristics with changes in rotational speed, mass eccentricity of rotor, sealing clearance, and sealing pressure drop at a specific seal fractional order. Cheng et al. [23] investigated the dynamic properties of a rotor-bearing system with different compliances, radial clearances, rotor-stator rubbing, raceway defects, and surface waviness in an analytical model. Dynamic studies focusing on rotor-bearing systems have shown that faults occurring in the bearings will cause significant changes in rotor dynamic characteristics, which will affect the distribution of the air gap between the stator and rotor in the motor. A nonuniform and varying air gap will not only introduce the effect of UMP but will also affect the inductance between the stator and rotor, which will also affect the UMP through the current. Therefore, we believe that the modeling and fault characteristics study for motor bearings should be based on a bidirectional coupling of dynamic and electromagnetic models. Currently, only a few researchers have conducted studies on electromagnetic-dynamic coupled modeling for induction motors. Fourati et al. [24] proposed an electromagnetic-dynamic model to describe the dynamical behavior of an elastically supported rotating shaft coupled to a squirrel-cage induction motor. Han et al. [25] proposed an electromagnetic-dynamic coupling model based on magnetic equivalent circuits, which can consider nonlinear air gap permeance, nonlinear iron material, and magnetic saturation. The main difference between the models developed by the above studies is the choice of the induction motor electromagnetic model, which may produce differences in computational speed and accuracy. However, the existing coupled model only considers the unidirectional coupling from the dynamic model to the electromagnetic model, which may bring some unexplained simulation biases. Therefore, in order to investigate the effect of whether to consider the UMP on the simulation results in the coupled model, we propose an electromagnetic-dynamic coupled modeling method considering the UMP. UMP is introduced into the coupled model, which is based on rotor-bearing dynamic model and multiple coupled circuit (MCC) theory for the first time. The dynamic response of the coupled model is solved by the numerical iterative algorithm.

This paper is organized as follows. Section 2 briefly describes the modeling process of the coupled model, containing the theoretical formulations of the rotor-bearing dynamic model and the MCC model, the calculation methods of inductance and UMP, and the overall logic of the model. Section 3 presents the simulation, experimental results, and analysis of the coupled model. Finally, conclusions are given in Section 4.

## 2. Electromagnetic-Dynamic Coupled Modeling Process Considering Unbalanced Magnetic Pull

### 2.1. Rotor-Bearing Dynamic Model

**Figure 1:**
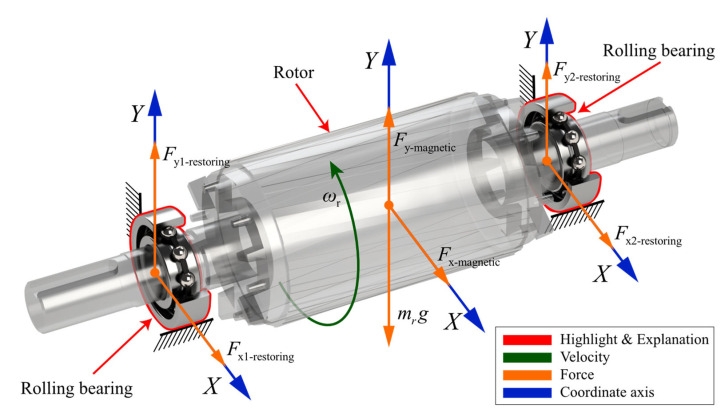
Rotor-bearing dynamic model.

In contrast to the signal model, the simulation results provided by the dynamic model are more objective in reflecting the real physical situation. In this work, the nonlinearities of the rotor-bearing dynamics model are considered by introducing the bearing clearance and the additional radial clearance due to spalling. As shown in Figure 1, the radial force supporting the rotor rotation is provided by the Hertzian contact force between the ball and the inner ring in the bearing. The UMP acts directly on the rotor.

The contact deformation of the *j*-th ball in the bearing is

(1) $$\delta_{j}=x\sin\theta_{j}+y\cos\theta_{j}-c_{l}-c_{lj}.$$

Han et al. [25] simulated the additional radial clearance generated when the ball passes through the defective position by the half-wave sine function:

(2) $$c_{lj}=\left\{\begin{array}{ll} h\sin\left(\frac{\pi }{\Delta \theta_{\mathrm{spall}}}\left(\theta_{j}-\theta_{\mathrm{spall}}\right)\right), & \mathrm{mod}\left(\theta_{j}-\theta_{\mathrm{spall}},2\pi \right)<\Delta \theta_{\mathrm{spall}}\\ 0, & \mathrm{else} \end{array}\right.,$$

(3) $$h=\left\{\begin{array}{cc} \left(\frac{d_{\mathrm{ball}}}{2}-\sqrt{{\left(\frac{d_{b}}{2}\right)}^{2}-{\left(\frac{w_{\mathrm{spall}}}{2}\right)}^{2}}\right)-\left(\frac{d_{o}}{2}-\sqrt{{\left(\frac{d_{o}}{2}\right)}^{2}-{\left(\frac{w_{\mathrm{spall}}}{2}\right)}^{2}}\right), & \mathrm{Outer}\;\mathrm{race}\;\mathrm{fault}\\ \left(\frac{d_{\mathrm{ball}}}{2}-\sqrt{{\left(\frac{d_{b}}{2}\right)}^{2}-{\left(\frac{w_{\mathrm{spall}}}{2}\right)}^{2}}\right)+\left(\frac{d_{i}}{2}-\sqrt{{\left(\frac{d_{i}}{2}\right)}^{2}-{\left(\frac{w_{\mathrm{spall}}}{2}\right)}^{2}}\right), & \mathrm{Inner}\;\mathrm{race}\;\mathrm{fault} \end{array}\right.,$$

(4) $$\Delta \theta_{\mathrm{spall}}=\begin{array}{c} 2\arcsin\left(\frac{w_{\mathrm{spall}}}{2R_{o}}\right) \end{array}.$$

According to the Hertzian contact theory, the total restoring force of the bearing in the X and Y directions is

(5) $$F_{x-\mathrm{restoring}}=\sum_{j=1}^{n_{\mathrm{ball}}}\lambda_{j}K_{c}\delta_{j}^{3/2}\sin\theta_{j},$$

(6) $$F_{y-\mathrm{restoring}}=\sum_{j=1}^{n_{\mathrm{ball}}}\lambda_{j}K_{c}\delta_{j}^{3/2}\cos\theta_{j},$$

$\lambda_{j}=1$ when $\delta_{j}>0$, otherwise, $\lambda_{j}=0$.

Finally, considering the effect of UMP, the differential equation for the vibration of the rotor-bearing system is formulated as

(7) $$m_{r}{\ddot{x}}_{r}+c_{r}{\dot{x}}_{r}+F_{x1-\mathrm{restoring}}+F_{x2-\mathrm{restoring}}+F_{x-\mathrm{magnetic}}=0,$$

(8) $$m_{r}{\ddot{y}}_{r}+c_{r}{\dot{y}}_{r}-F_{y1-\mathrm{restoring}}-F_{y2-\mathrm{restoring}}-F_{y-\mathrm{magnetic}}=-m_{r}g.$$

### 2.2. Electromagnetic Model Based on Multiple Coupled Circuit Theory

In industrial applications, motors are inevitably operated under asymmetric supply conditions, accompanied by saturation effect, eddy current loss, and friction loss. If the influence of the above factors can be effectively introduced into the process of motor model construction, a simulation model closer to the real motor could be obtained. However, the consideration of detailed factors always implies the growth of model parameters and complexity of the structure, which leads to small improvements in results and huge increases in costs. It is not compatible with the cost-efficiency ratio requirements of industrial applications. Furthermore, the objective in this study is to construct coupled models that can effectively reflect the fault transmission paths and generate usable fault signals, which can help to qualitatively analyze and explain the fault mechanisms. Therefore, several popular simplifying assumptions were used in the construction of the MCC model to help improve the computational efficiency. These assumptions are as follows.

1. The motor is powered by a balanced three-phase voltage source;
2. Hysteresis loss, eddy current loss, and friction loss are ignored;
3. Rotor bars are insulated from each other.

**Figure 2:**
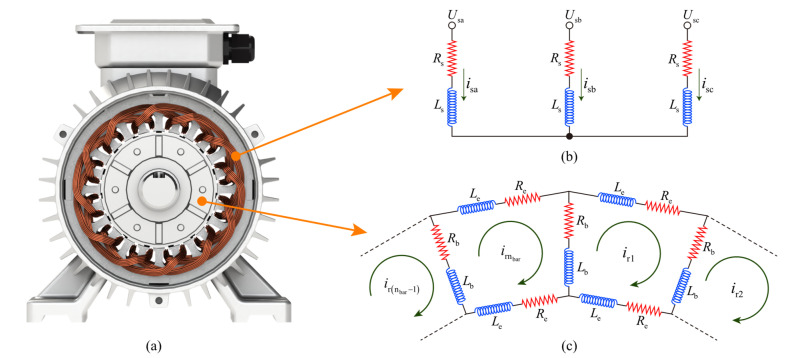
The stator and rotor are equivalent to an MCC model. (**a**) Induction motor; (**b**) stator equivalent circuit; (**c**) squirrel cage rotor equivalent circuit.

As shown in Figure 2, the MCC theory treats each stator winding phase as a series circuit of resistance and inductance and treats the squirrel-cage rotor as a loop evenly distributed in space. The voltage equation for a three-phase squirrel-cage induction motor when star-connected is

(9) $$U_{s}=I_{s}R_{s}+\frac{d}{dt}\lambda_{s},$$

(10) $$0=I_{r}R_{r}+\frac{d}{dt}\lambda_{r},$$

where $U_{s}=\left[\begin{array}{c} U_{\mathrm{sa}}\\ U_{\mathrm{sb}}\\ U_{\mathrm{sc}} \end{array}\right],I_{s}=\left[\begin{array}{c} i_{\mathrm{sa}}\\ i_{\mathrm{sb}}\\ i_{\mathrm{sc}} \end{array}\right],R_{s}=\left[\begin{array}{ccc} R_{s} & 0 & 0\\ 0 & R_{s} & 0\\ 0 & 0 & R_{s} \end{array}\right],\lambda_{s}=\left[\begin{array}{c} \lambda_{\mathrm{sa}}\\ \lambda_{\mathrm{sb}}\\ \lambda_{\mathrm{sc}} \end{array}\right]$,

$$R_{r}=\left[\begin{array}{cccccc} 2\left(R_{b}+R_{e}\right) & -R_{b} & 0 & \cdots & -R_{b} & -R_{e}\\ -R_{b} & 2\left(R_{b}+R_{e}\right) & -R_{b} & \cdots & 0 & -R_{e}\\ 0 & -R_{b} & 2\left(R_{b}+R_{e}\right) & \cdots & 0 & -R_{e}\\ \vdots & \vdots & \vdots & \vdots & \vdots & \vdots \\ -R_{b} & 0 & 0 & \cdots & 2\left(R_{b}+R_{e}\right) & -R_{e}\\ -R_{e} & -R_{e} & -R_{e} & \cdots & -R_{e} & n_{\mathrm{bar}}R_{e} \end{array}\right],i_{r}=\left[\begin{array}{c} i_{r1}\\ i_{r2}\\ i_{r3}\\ \vdots \\ i_{rn_{\mathrm{bar}}}\\ i_{e} \end{array}\right],\lambda_{r}=\left[\begin{array}{c} \lambda_{r1}\\ \lambda_{r2}\\ \lambda_{r3}\\ \vdots \\ \lambda_{rn_{\mathrm{bar}}}\\ \lambda_{e} \end{array}\right].$$

The flux linkage equation of stator and rotor is

(11) $$\lambda_{s}=L_{\mathrm{ss}}I_{s}+L_{\mathrm{sr}}I_{r},$$

(12) $$\lambda_{r}=L_{\mathrm{rs}}I_{s}+L_{\mathrm{rr}}I_{r}=L_{\mathrm{sr}}^{T}I_{s}+L_{\mathrm{rr}}I_{r},$$

where $L_{\mathrm{rs}}=L_{\mathrm{sr}}^{T}$, $L_{\mathrm{ss}}=\left[\begin{array}{ccc} L_{\mathrm{sasa}} & L_{\mathrm{sasb}} & L_{\mathrm{sasc}}\\ L_{\mathrm{sbsa}} & L_{\mathrm{sbsb}} & L_{\mathrm{sbsc}}\\ L_{\mathrm{scsa}} & L_{\mathrm{scsb}} & L_{\mathrm{scsc}} \end{array}\right]$, $L_{\mathrm{sr}}=\left[\begin{array}{cccccc} L_{\mathrm{sar}1} & L_{\mathrm{sar}2} & L_{\mathrm{sar}3} & \cdots & L_{\mathrm{sar}n_{\mathrm{bar}}} & 0\\ L_{\mathrm{sbr}1} & L_{\mathrm{sbr}2} & L_{\mathrm{sbr}3} & \cdots & L_{\mathrm{sbr}n_{\mathrm{bar}}} & 0\\ L_{\mathrm{scr}1} & L_{\mathrm{scr}2} & L_{\mathrm{scr}3} & \cdots & L_{\mathrm{scr}n_{\mathrm{bar}}} & 0 \end{array}\right]$,

$$L_{r}=\left[\begin{array}{cccccc} L_{r1r1}+2\left(L_{b}+L_{e}\right) & L_{r1r2}-L_{b} & L_{r1r3} & \cdots & L_{r1rn_{\mathrm{bar}}}-L_{b} & -L_{e}\\ L_{r2r1}-L_{b} & L_{r2r2}+2\left(L_{b}+L_{e}\right) & L_{r2r3}-L_{b} & \cdots & L_{r2rn_{\mathrm{bar}}} & -L_{e}\\ L_{r3r1} & L_{r3r2}-L_{b} & L_{r3r3}+2\left(L_{b}+L_{e}\right) & \cdots & L_{r3rn_{\mathrm{bar}}} & -L_{e}\\ \vdots & \vdots & \vdots & \vdots & \vdots & \vdots \\ L_{rn_{\mathrm{bar}}r1}-L_{b} & L_{rn_{\mathrm{bar}}r2} & L_{rn_{\mathrm{bar}}r3} & \cdots & L_{rn_{\mathrm{bar}}rn_{\mathrm{bar}}}+2\left(L_{b}+L_{e}\right) & -L_{e}\\ -L_{e} & -L_{e} & -L_{e} & \cdots & -L_{e} & n_{\mathrm{bar}}L_{e} \end{array}\right]$$.

The electromagnetic torque equation in matrix form:

(13) $$\begin{array}{c} T_{e}=\frac{1}{2}\frac{p}{2}{\left[\begin{array}{c} I_{s}\\ I_{r} \end{array}\right]}^{T}\frac{d}{d\theta_{e}}\left[\begin{array}{cc} L_{\mathrm{ss}} & L_{\mathrm{sr}}\\ L_{\mathrm{rs}} & L_{\mathrm{rr}} \end{array}\right]\left[\begin{array}{c} I_{s}\\ I_{r} \end{array}\right]\\ =\frac{1}{2}\frac{p}{2}\left(I_{s}^{T}\frac{dL_{\mathrm{ss}}}{d\theta_{e}}I_{s}+I_{s}^{T}\frac{dL_{\mathrm{sr}}}{d\theta_{e}}I_{r}+I_{r}^{T}\frac{dL_{\mathrm{rs}}}{d\theta_{e}}I_{s}+I_{r}^{T}\frac{dL_{\mathrm{rr}}}{d\theta_{e}}I_{r}\right) \end{array}$$

The motor dynamic equation:

(14) $$T_{e}=J\frac{d\omega_{\mathrm{rm}}}{dt}+T_{l}.$$

### 2.3. Calculation of Inductance and Unbalanced Magnetic Pull

**Figure 3:**
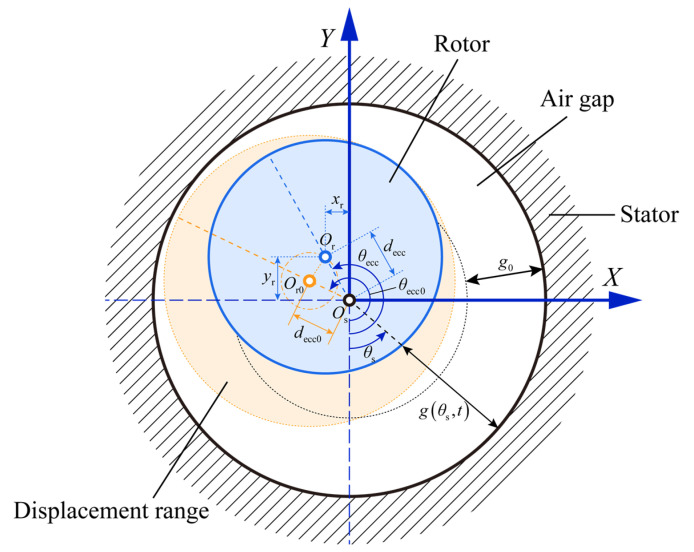
Nonuniform distribution of air gap caused by rotor eccentricity.

The rotor radial displacement determines the air gap distribution between the stator and rotor, as shown in Figure 3. Strictly speaking, the air gap in real conditions is always nonuniform due to manufacturing errors and gravity, etc., which is aggravated by the additional radial displacements caused by bearing faults. The bearing faults cause the dynamic eccentricity of the rotor due to the variation of the restoring force, so as to obtain the time-varying air gap. The distribution of the air gap can be calculated in successive numerical iterations. According to the displacement of the rotor in the x and y directions at moment t, the rotor eccentricity, eccentricity angle, and relative eccentricity introduced by the bearing faults can be obtained:

(15) $$d_{\mathrm{ecc}}=\sqrt{x_{r}^{2}+y_{r}^{2}},$$

(16) $$\theta_{\mathrm{ecc}}=\left\{\begin{array}{ll} \arccos\left(-\frac{y_{r}}{d_{\mathrm{ecc}}}\right), & x_{r}\geq 0\\ 2\pi -\arccos\left(-\frac{y_{r}}{d_{\mathrm{ecc}}}\right), & x_{r}<0 \end{array}\right.,$$

(17) $$e=\frac{d_{\mathrm{ecc}}}{g_{0}}.$$

Hence, the distribution of the air gap length at moment t is

(18) $$g\left(\theta_{s},t\right)=g_{0}-d_{\mathrm{ecc}}\cos\left(\theta_{\mathrm{ecc}}-\theta_{s}\right).$$

The air gap length distribution is associated with the inductance through the winding function approach (WFA), which in turn affects the stator current in the MCC model. The WFA allows one to calculate the time-varying inductance between the stator and rotor by the motor winding structure and the air gap length distribution. The assumptions when applying the winding function method are as follows.

1. The flux linkage passes through the air gap radially, that is, the axial flux linkage is ignored;
2. The magnetic permeability of magnetic materials is infinite;
3. There is a negligible slot effect.

By derivation based on Gauss’s law and Ampere’s law, the equation for the WFA to calculate the mutual inductance between any coil $c_{1}$ and coil $c_{2}$ can be expressed as

(19) $$L_{c_{1}c_{2}}=\mu_{0}rl\int_{0}^{2\pi }n_{c_{1}}\left(\theta_{s}\right)\cdot N_{c_{2}}\left(\theta_{s}\right)\cdot g^{-1}\left(\theta_{s}\right)d\theta_{s},$$

where the winding function $N_{c_{2}}\left(\theta_{s}\right)$ is a function of the turn function $n_{c_{1}}\left(\theta_{s}\right)$ and the air gap length $g\left(\theta_{s}\right)$:

(20) $$N_{c_{2}}\left(\theta_{s}\right)=n_{c_{2}}\left(\theta_{s}\right)-\frac{1}{2\pi \langle g^{-1}\left(\theta_{s}\right)\rangle }\int_{0}^{2\pi }n_{c_{2}}\left(\theta_{s}\right)\cdot g^{-1}\left(\theta_{s}\right)d\theta_{s}.$$

Taking into account the relative motion between the stator and rotor, the WFA equation can also be rewritten as a function of the rotor’s mechanical angular position or time t:

(21) $$L_{c_{1}c_{2}}\left(\theta_{r}\right)=\mu_{0}rl\int_{0}^{2\pi }n_{c_{1}}\left(\theta_{s},\theta_{r}\right)\cdot N_{c_{2}}\left(\theta_{s},\theta_{r}\right)\cdot g^{-1}\left(\theta_{s},\theta_{r}\right)d\theta_{s},$$

(22) $$L_{c_{1}c_{2}}\left(t\right)=\mu_{0}rl\int_{0}^{2\pi }n_{c_{1}}\left(\theta_{s},t\right)\cdot N_{c_{2}}\left(\theta_{s},t\right)\cdot g^{-1}\left(\theta_{s},t\right)d\theta_{s}.$$

The reason of UMP is that the nonuniform air gap generated by the eccentricity makes the flux density unevenly distributed. The presence of UMP tends to pull the rotor in an eccentric direction, which can lead to more complex rotor dynamics. Therefore, many studies have focused on the analytical calculation of the UMP. Based on the expression for the UMP proposed by Guo et al. [16], Zhou et al. [26] derived the general expression for the UMP at different number of poles:

(23) $$F_{x-\mathrm{magnetic}}=\left\{\begin{array}{cc} \begin{array}{c} f_{1}\cos\left(\theta_{\mathrm{ecc}}\right)+f_{2c}\cos\left(2\omega_{\mathrm{re}}t-\theta_{\mathrm{ecc}}\right)+f_{2s}\sin\left(2\omega_{\mathrm{re}}t-\theta_{\mathrm{ecc}}\right)+\\ f_{3c}\cos\left(2\omega_{\mathrm{re}}t-3\theta_{\mathrm{ecc}}\right)+f_{3s}\sin\left(2\omega_{\mathrm{re}}-3\theta_{\mathrm{ecc}}\right), \end{array} & p=2\\ \begin{array}{c} f_{1}\cos\left(\theta_{\mathrm{ecc}}\right)+f_{3c}\cos\left(2\omega_{\mathrm{re}}t-3\theta_{\mathrm{ecc}}\right)+f_{3s}\sin\left(2\omega_{\mathrm{re}}t-3\theta_{\mathrm{ecc}}\right)+\\ f_{4c}\cos\left(2\omega_{\mathrm{re}}t-5\theta_{\mathrm{ecc}}\right)+f_{4s}\sin\left(2\omega_{\mathrm{re}}t-5\theta_{\mathrm{ecc}}\right), \end{array} & p=4\\ f_{1}\cos\left(\theta_{\mathrm{ecc}}\right)+f_{4c}\cos\left(2\omega_{\mathrm{re}}t-5\theta_{\mathrm{ecc}}\right)+f_{4s}\sin\left(2\omega_{\mathrm{re}}t-5\theta_{\mathrm{ecc}}\right), & p=6\\ f_{1}\cos\left(\theta_{\mathrm{ecc}}\right), & p\geq 8 \end{array}\right.$$

(24) $$F_{y-\mathrm{magnetic}}=\left\{\begin{array}{cc} \begin{array}{c} f_{1}\sin\left(\theta_{\mathrm{ecc}}\right)+f_{2c}\sin\left(2\omega_{\mathrm{re}}t-\theta_{\mathrm{ecc}}\right)-f_{2s}\cos\left(2\omega_{\mathrm{re}}t-\theta_{\mathrm{ecc}}\right)-\\ f_{3c}\sin\left(2\omega_{\mathrm{re}}t-3\theta_{\mathrm{ecc}}\right)+f_{3s}\cos\left(2\omega_{\mathrm{re}}t-3\theta_{\mathrm{ecc}}\right), \end{array} & p=2\\ \begin{array}{c} f_{1}\sin\left(\theta_{\mathrm{ecc}}\right)+f_{3c}\sin\left(2\omega_{\mathrm{re}}t-3\theta_{\mathrm{ecc}}\right)-f_{3s}\cos\left(2\omega_{\mathrm{re}}t-3\theta_{\mathrm{ecc}}\right)-\\ f_{4c}\sin\left(2\omega_{\mathrm{re}}t-5\theta_{\mathrm{ecc}}\right)+f_{4s}\cos\left(2\omega_{\mathrm{re}}t-5\theta_{\mathrm{ecc}}\right), \end{array} & p=4\\ f_{1}\sin\left(\theta_{\mathrm{ecc}}\right)+f_{4c}\sin\left(2\omega_{\mathrm{re}}-5\theta_{\mathrm{ecc}}\right)-f_{4s}\cos\left(2\omega_{\mathrm{re}}-5\theta_{\mathrm{ecc}}\right), & p=6\\ f_{1}\sin\left(\theta_{\mathrm{ecc}}\right), & p\geq 8 \end{array}\right.$$

where:

$$\omega_{\mathrm{re}}=\omega_{\mathrm{rm}}\frac{p}{2},$$

$$\begin{array}{cc} \; & f_{1}=\frac{RL\pi }{4\mu_{0}}\left(2A_{0}A_{1}+A_{1}A_{2}+A_{2}A_{3}\right)\times \left(F_{s}^{2}+F_{r}^{2}+2F_{s}F_{r}\cos\varphi_{\mathrm{sr}}\right)\\ \; & f_{2c}=\frac{RL\pi }{4\mu_{0}}\left(A_{0}A_{1}+\frac{1}{2}A_{1}A_{2}+\frac{1}{2}A_{2}A_{3}\right)\times \left(F_{s}^{2}+2F_{s}F_{r}\cos\varphi_{\mathrm{sr}}+F_{r}^{2}\cos2\varphi_{\mathrm{sr}}\right),\\ \; & f_{2s}=\frac{RL\pi }{4\mu_{0}}\left(A_{0}A_{1}+\frac{1}{2}A_{1}A_{2}+\frac{1}{2}A_{2}A_{3}\right)\times \left(2F_{s}F_{r}\sin\varphi_{\mathrm{sr}}+F_{r}^{2}\sin2\varphi_{\mathrm{sr}}\right),\\ \; & f_{3c}=\frac{RL\pi }{4\mu_{0}}\left(A_{0}A_{3}+\frac{1}{2}A_{1}A_{2}\right)\times \left(F_{s}^{2}+2F_{s}F_{r}\cos\varphi_{\mathrm{sr}}+F_{r}^{2}\cos2\varphi_{\mathrm{sr}}\right),\\ \; & f_{3s}=\frac{RL\pi }{4\mu_{0}}\left(A_{0}A_{3}+\frac{1}{2}A_{1}A_{2}\right)\times \left(2F_{s}F_{r}\sin\varphi_{\mathrm{sr}}+F_{r}^{2}\sin2\varphi_{\mathrm{sr}}\right),\\ \; & f_{4c}=\frac{RL\pi }{8\mu_{0}}A_{2}A_{3}\times \left(F_{s}^{2}+2F_{s}F_{r}\cos\varphi_{\mathrm{sr}}+F_{r}^{2}\cos2\varphi_{\mathrm{sr}}\right),\\ \; & f_{4s}=\frac{RL\pi }{8\mu_{0}}A_{2}A_{3}\times \left(2F_{s}F_{r}\sin\varphi_{\mathrm{sr}}+F_{r}^{2}\sin2\varphi_{\mathrm{sr}}\right) \end{array}$$

$$A_{n}=\left\{\begin{array}{ll} \frac{\mu_{0}}{g_{0}}\frac{1}{\sqrt{1-e^{2}}}, & n=0\\ \frac{2\mu_{0}}{g_{0}}\frac{1}{\sqrt{1-e^{2}}}{\left(\frac{1-\sqrt{1-e^{2}}}{e}\right)}^{n}, & n>0 \end{array}\right..$$

**Figure 4:**
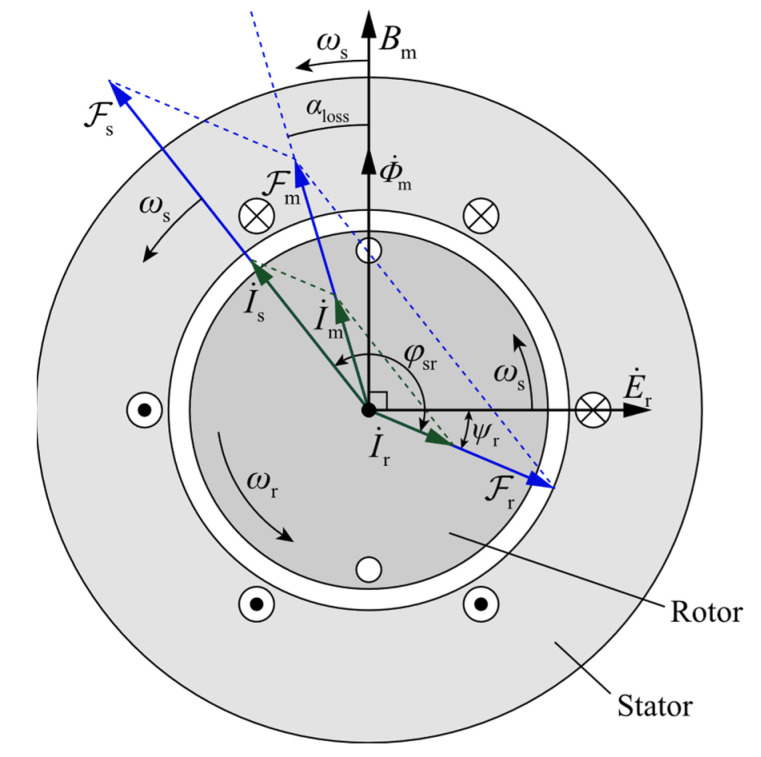
Synthesis of stator and rotor magnetomotive force.

Figure 4 shows the synthesis of the stator magnetomotive force (MMF) and rotor magnetomotive force when the motor is running with a load. Due to the assumption that hysteresis loss and eddy loss are not considered in the MCC model, the core loss angle $\alpha_{\mathrm{loss}}=0^{\circ }$. The rotor leakage reactance causes the rotor MMF to lag behind the induced electromotive force by an impedance angle $\psi_{r}$. When the rotor leakage reactance is small, $\psi_{r}\approx 0$, and thus, $\varphi_{\mathrm{sr}}=\pi -\arccos\left(\frac{F_{r}}{F_{s}}\right)$.

The stator and rotor MMF amplitudes are calculated by:

(25) $$F=0.9\times \frac{n_{\mathrm{phase}}}{2}\times \frac{Nk_{w}I_{\mathrm{rms}}}{p}.$$

The object of this paper is a 3-phase 2-pole squirrel-cage induction motor, whose stator windings are distributed in a single-layer and full-pitch manner. For the stator, the number of phases $n_{\mathrm{phase}}=3$, the total number of series turns per phase for single-layer winding distribution $N=\frac{p}{2}Q_{s}Z_{s}$, and the fundamental winding factor $k_{w}=k_{p}k_{d}=k_{p}\frac{\sin\left(Q_{s}\alpha /2\right)}{Q_{s}\sin\left(\alpha /2\right)}$, where $k_{p}=1$ and $\alpha =\frac{p}{2}\cdot \frac{2\pi }{Q_{s}}$. For the rotor, the number of phases $n_{\mathrm{phase}}=\frac{Q_{r}}{p/2}$, the total number of series turns per phase $N=\frac{1}{2}$, and the fundamental winding factor $k_{w}=1$.

### 2.4. Electromagnetic-Dynamic Coupled Model Framework

**Figure 5:**
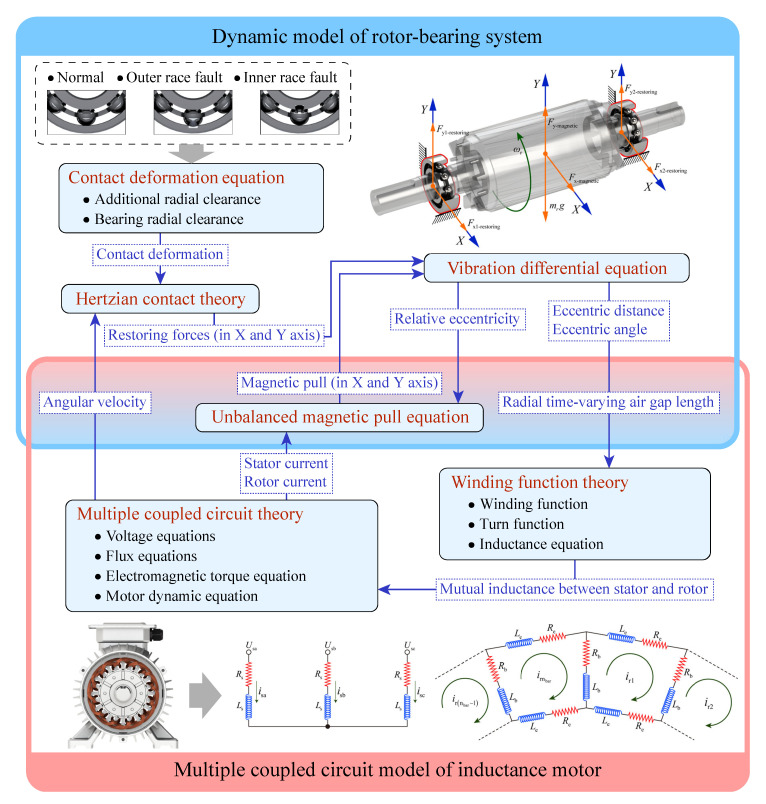
Framework of electromagnetic-dynamic coupled model with consideration of unbalanced magnetic pull.

As shown in Figure 5, the bidirectional coupling between the rotor-bearing system dynamic model and the induction motor MCC model is achieved by the variables in the center coloring area. The blue frame represents the dynamic model of the rotor-bearing system, and the red frame represents the MCC model of the induction motor. In the dynamic model, the contact deformation equation includes a preset bearing fault state. Based on the Hertzian contact theory, the restoring force of the bearing in the X and Y directions can be calculated from the contact deformation and the ball position obtained by integrating the angular velocity. Since the UMP and restoring forces act on the rotor simultaneously, both are introduced into the vibration differential equation. The air gap length distribution is related to the stator–rotor inductance by the WFA, and it can be obtained by solving the vibration differential equation of the rotor-bearing system. The inductance between the stator and rotor directly affects the state of the motor MCC model and is reflected in the system’s state parameters such as current and rotor angular velocity. The rotor angular velocity is fed back to the calculation of the bearing restoring force through the Hertzian contact theory, which is one of the paths by which the electromagnetic model affects the dynamic model. In addition, regarding the UMP calculated by current, relative eccentricity will be fed back to the vibration differential equation, potentially causing more complex rotor dynamic behavior.

## 3. Simulation, Experimentation, and Analysis of Results

### 3.1. Model Parameters and Experimental Conditions

The parameters in the coupled model are set according to the 3-phase 2-pole induction motor used in the experiments, as shown in Table 1 and Table 2. Due to the limitations of measurement means and methods, a few mechanical and electrical parameters that were difficult to determine were estimated with reference to parameters from other works of the literature. The faults in the inner and outer races of the bearings are spalling faults with the same width. In order to highlight the bearing fault characteristics, the spalling position of the outer race fault is set directly below the inside of the outer race. Because of the complex rotational state of the ball in the raceway, this paper will not discuss the ball spalling fault in the bearing for the time being.

**Figure 6:**
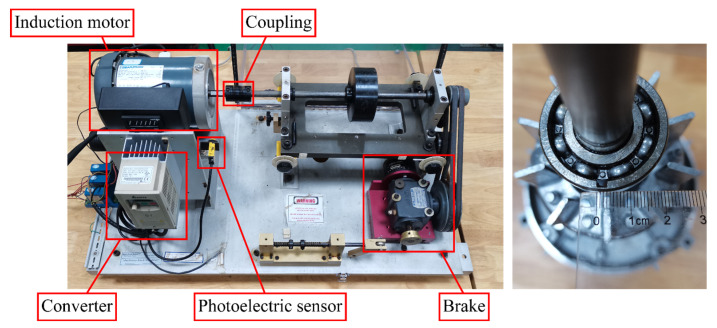
Mechanical fault simulation experimental bench and fault bearing.

The experimental bench and bearings used to simulate the faults are shown in Figure 6. The vibration signal on the motor end shield is measured by an IMI 608A11 accelerometer. The inverter provides a three-phase voltage at a specific frequency to the induction motor. The photoelectric sensor collects the pulse signal reflecting the rotational velocity by detecting the reflective strip located on the rotating shaft. The brake is connected to the induction motor via a rotor shaft and coupling to simulate the load at work. The bearing outer race spalling fault is simulated by cutting grooves on the inside of the outer race with a spalling width of about 3 mm, as shown in the Figure 6. Due to the low number of motor poles, this experiment was conducted at 20 Hz supply frequency for safety reasons.

### 3.2. Vibration Characteristics Analysis

**Figure 7:**
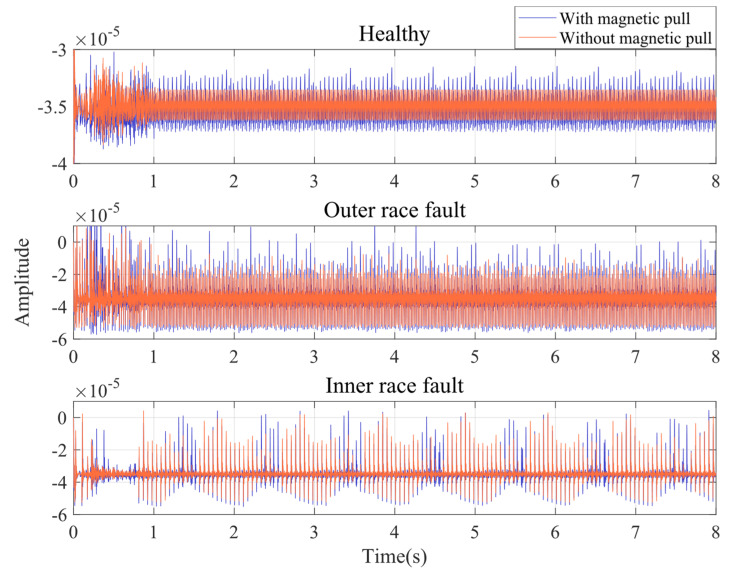
Time domain signal of rotor displacement in Y direction.

This is performed for the purpose of studying the effect of UMP on the rotor dynamic behavior. The simulation time is set to 8 s, and the coupled model is used to simulate the normal, outer race fault, and inner race fault states of the induction motor in two cases: without magnetic pull and with magnetic pull. The rotor vibration time domain in Figure 7 shows the effect of whether or not UMP is introduced. The simulation results show that the healthy motor rotor has stationary vibration without UMP. In the presence of UMP, the vibration amplitude of the healthy motor rotor increases, and modulation occurs. The spalling located on the outer race of the bearing makes the rotor vibration waveform have regular shocks, while the presence of UMP seems to make the shock amplitude vary irregularly. In case of inner race faults, there is a significant amplitude modulation in the vibration waveform without UMP. With the introduction of the UMP, the amplitude in the originally low amplitude range (formed by amplitude modulation) increases significantly. In general, the effect of UMP on rotor vibration in the time domain is mainly shown as an increase in amplitude and jitter.

**Figure 8:**
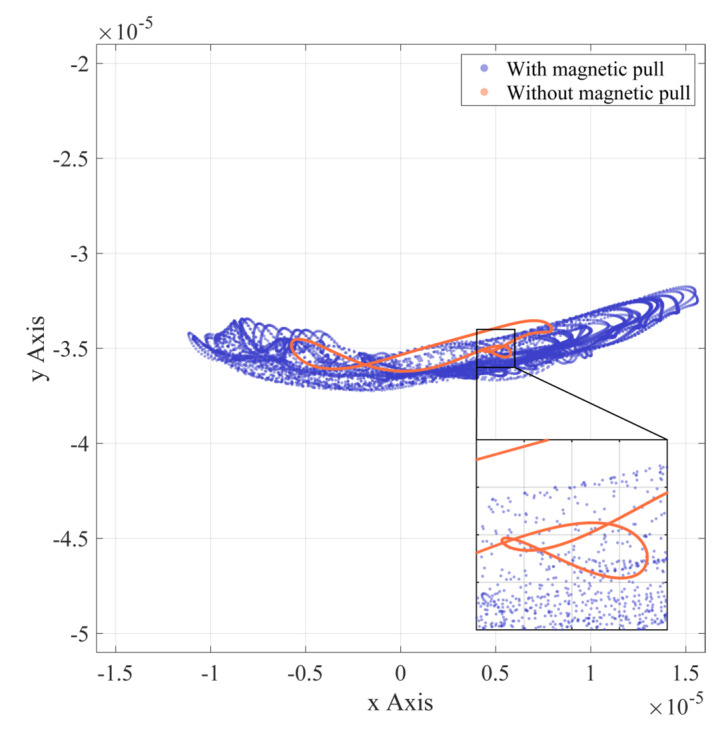
Rotor axis trajectory (normal).

**Figure 9:**
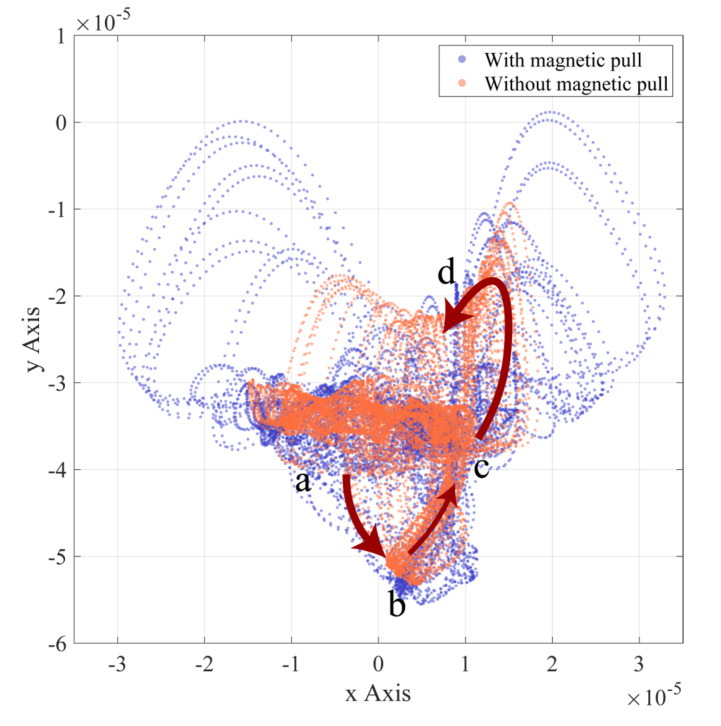
Rotor axis trajectory (outer race fault).

**Figure 10:**
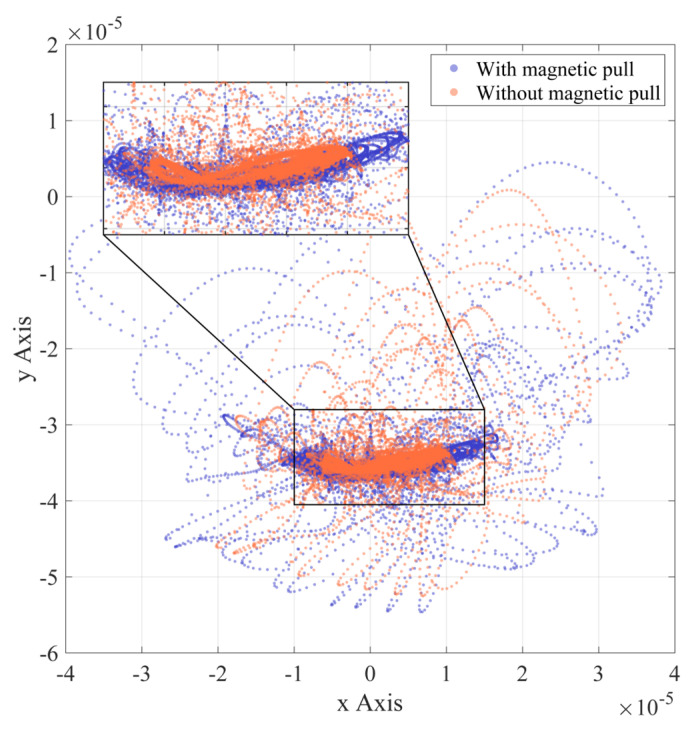
Rotor axis trajectory (inner race fault).

**Figure 11:**
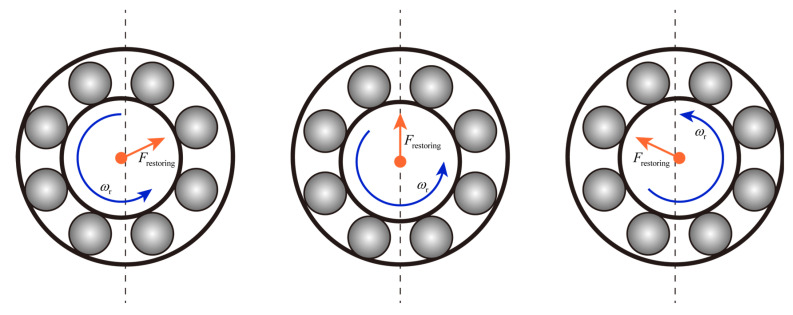
Changes in restoring forces due to bearing radial clearance and rotor position.

Figure 8, Figure 9 and Figure 10 show the rotor axis trajectories for 7~8 s in normal, outer race fault, and inner race fault states, respectively. Under normal conditions, rotor displacement exists mainly in the horizontal direction, which may be due to bearing radial clearance. As shown in Figure 11, the radial clearance allows only a portion of the balls to be in contact with both the inner and outer races, so not all of the balls provide restoring force. The component of the combined restoring force in the horizontal direction depends on the rotor position and fluctuates with the rotor position. At the same time, the vertical vibration of the rotor caused by the restoring force fluctuation is limited by the presence of gravity. As shown in Figure 8, in the absence of UMP, the rotor axis has a clear trajectory despite the nonlinear factor caused by the radial clearance. When there is UMP, the range of the axis trajectory is significantly expanded, and the shape is close to the irregular motion under the condition of no UMP.

**Figure 12:**
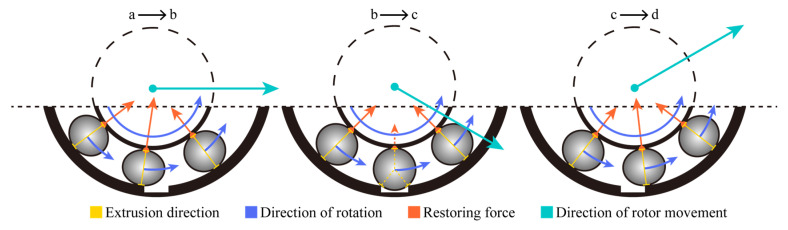
Rolling ball moves in and out of the bearing defective position.

In the axial trajectory for the bearing outer race fault condition shown in Figure 9, the defect located at the bottom of the outer race causes the axial displacement in the vertical direction. The density of the axial trajectory reflects the difference in speed of the ball as it moves in and out of the defect position during counterclockwise rotation. The analysis of the process from position a to position d in Figure 9 is shown in Figure 12. As the deformation of the ball entering the defective position gradually decreases, the bearing restoring force supporting the rotor in the vertical direction gradually decreases. With gravity, the rotor accelerates from position a to position b. Position b to c is the process of the ball leaving the defective position. In this process, the ball deformation increases and the vertical restoring force increases. The resultant forces of the vertical restoring force and gravity support the rotor to accelerate upward after preventing it from continuing to fall. Due to the process of counteracting the falling momentum of the rotor, the rotor rises at a lower speed when the balls start to leave the defect position. In addition, before the ball enters the defect position, the resultant force of the bearing restoring force has a rightward horizontal component, and the rotor has a rightward horizontal velocity component. As the ball rotates counterclockwise away from the defect, although the rotor will rise to the vertical position before entering the defect, the speed of the rotor horizontally to the right at this time will tend to compress the balls leaving the defect. As a result, a greater restoring force of the ball leaving the defective position acts on the rotor and produces a behavior similar to “ejection“, forming an approximately parabolic axial trajectory from position c to d in Figure 9. The range of axial trajectories is extended by adding UMP, which may be due to the fact that UMP always tends to pull the rotor away from the equilibrium position.

As shown in Figure 10, when the bearing has an inner race fault, the balls can move in and out of the defective position at almost any angle because the spalled inner race rotates with the rotor. Therefore, a dynamic behavior similar to that described above for the outer race failure case can occur at all angles. Depending on the defect position angle, the specific behavior may vary in part, as reflected in the more haphazard parabolic-like trajectory in Figure 10. It is worth noting that the overall shape in the dense area of the axial trajectory is close to the trajectory in the normal case, regardless of whether UMP is introduced.

Summarizing the rotor motion trajectories in the three bearing states, the simulation results of introducing UMP often cause the expansion in displacement range and the increase in disorder.

**Figure 13:**
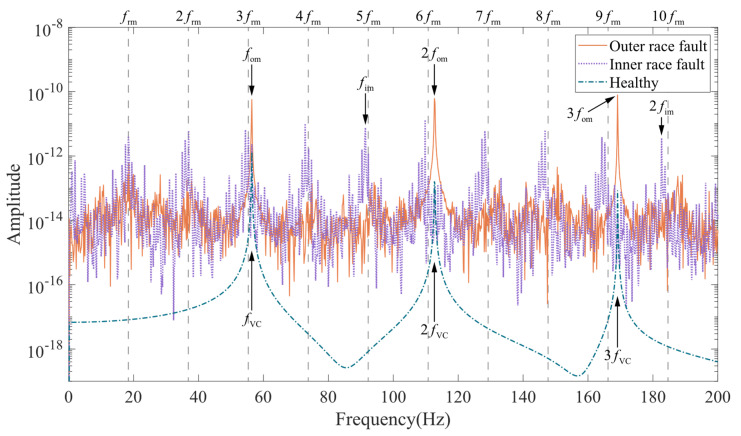
Rotor displacement spectrum in Y direction without unbalanced magnetic pull.

**Figure 14:**
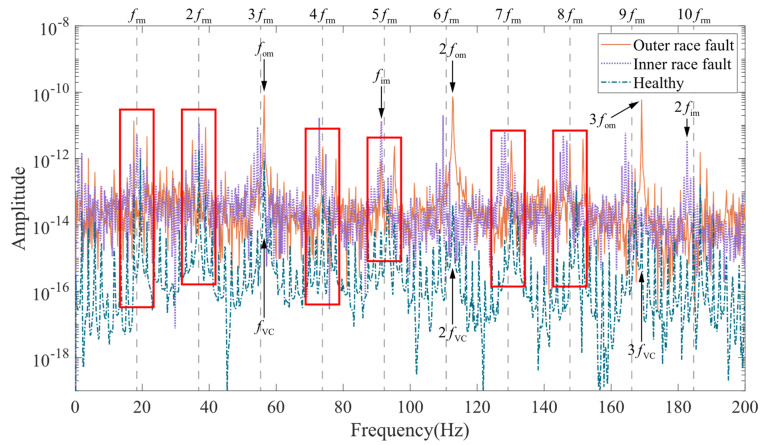
Rotor displacement spectrum in Y direction with unbalanced magnetic pull.

Frequency domain analysis can provide more information. For the SKF 6203 bearing used in the model, its outer race fault frequency is $f_{\mathrm{om}}=\frac{n_{\mathrm{ball}}}{2}f_{\mathrm{rm}}\left(1-\frac{d_{b}}{D_{b}}\cos\beta \right)=3.0681f_{\mathrm{rm}}$. The inner race fault frequency is $f_{\mathrm{im}}=\frac{n_{b}}{2}f_{\mathrm{rm}}\left(1+\frac{d_{b}}{D_{b}}\cos\beta \right)=4.9319f_{\mathrm{rm}}$. Figure 13 shows the simulated displacement spectrum of the rotor in the Y direction without UMP. The rotor displacement spectrum curve in the case of healthy bearings is smooth and has prominent amplitudes only at the variable compliance frequency ($f_{\mathrm{VC}}$, equal to the outer race fault frequency $f_{\mathrm{om}}\approx 56.43\mathrm{Hz}$) and its multiples ($2f_{\mathrm{VC}},3f_{\mathrm{VC}},\cdots$). In the case of outer race fault, the amplitude at the outer race fault frequency ($f_{\mathrm{om}}$) and its multiples ($2f_{\mathrm{om}},3f_{\mathrm{om}},\cdots$) increase significantly, while the amplitude on the other frequency components is also higher than that of the healthy bearing case. The inner race fault spectrum is significantly modulated by the rotational speed. In the marked red area, it can be seen that besides the inner race fault frequency ($f_{\mathrm{im}}$) and its multiples ($2f_{\mathrm{im}},\cdots$), the spectrum has the same prominent amplitude at the rotational frequency ($f_{\mathrm{rm}}$) and its multiples ($2f_{\mathrm{rm}},3f_{\mathrm{rm}},\cdots$). With the addition of UMP, the vibration spectrum of the coupled model showed a more significant change. As shown in the areas highlighted in red in Figure 14, the speed modulation with the presence of UMP appears in the spectrum for the healthy and outer race fault cases. The spectrum of the inner race fault is less affected.

**Figure 15:**
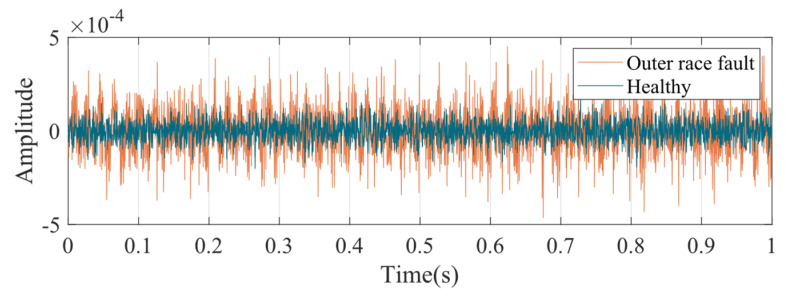
Time domain signal of acceleration at the drive end.

**Figure 16:**
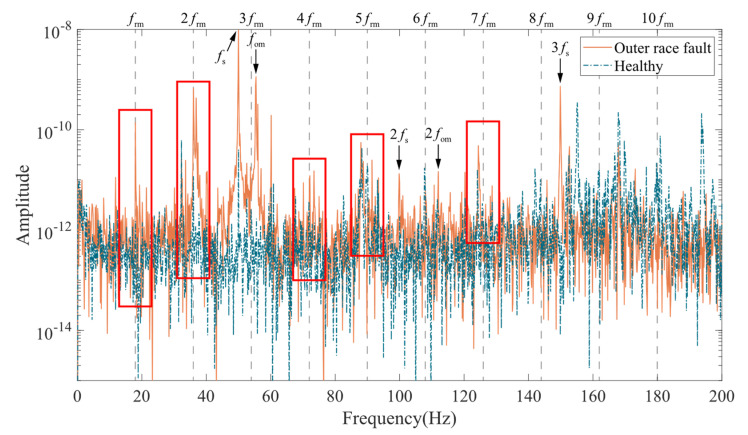
Acceleration spectrum at the drive end.

Experiments were conducted in the experimental bench in Figure 6, and the acceleration signal of the end shield on the motor drive end was measured. Figure 15 shows the time domain signal of motor end shield acceleration with outer race faulty bearing and normal bearing. Obviously, the shocks caused by the bearing faults make the peak acceleration in the fault condition higher than normal. From the perspective of frequency domain, the bearing outer race fault frequency ($f_{\mathrm{om}}$) and its multiples ($2f_{\mathrm{om}},3f_{\mathrm{om}},\cdots$) can be seen in the vibration spectrum of the end shield at the drive end shown in Figure 16. The peaks in the spectrum at the industrial frequency ($f_{s}$) and its multiples ($2f_{s},3f_{s},\cdots$) may be caused by poor grounding. It is worth noting that the spectral curves in the case of outer race fault have significant peaks at $f_{\mathrm{rm}}$,$2f_{\mathrm{rm}}$,$3f_{\mathrm{rm}}$, and $4f_{\mathrm{rm}}$, which may correspond to the speed modulation caused by the UMP in Figure 14. In addition, the average amplitude located in the red area is slightly higher than the surrounding area, which is also similar to the spectral pattern in Figure 14.

### 3.3. Current Characteristics Analysis

From the model framework shown in Figure 5, it can be seen that the rotor-bearing dynamic model affects the stator–rotor inductance through the air gap, which in turn affects the MCC model. Therefore, air gap changes due to bearing faults may be reflected in the stator current by inductance, making it possible to diagnose motor bearing faults by stator current. Schoen et al. [27] first proposed that bearing faults affect the stator current through the air gap and generate vibration frequency-related frequency components in the stator current. Blodt et al. [28] theoretically derived the equation for the characteristic frequency of bearing faults reflected in the stator current through the air gap.

- Outer race fault:
  (26) $$f_{\mathrm{oe}}=kf_{\mathrm{om}}\pm f_{s}.$$
- Inner race fault:
  (27) $$f_{\mathrm{ie}}=kf_{\mathrm{im}}\pm f_{s}\pm f_{\mathrm{rm}}.$$
- Ball fault:
  (28) $$f_{\mathrm{ie}}=kf_{\mathrm{im}}\pm f_{s}\pm f_{\mathrm{rm}}.$$

**Figure 17:**
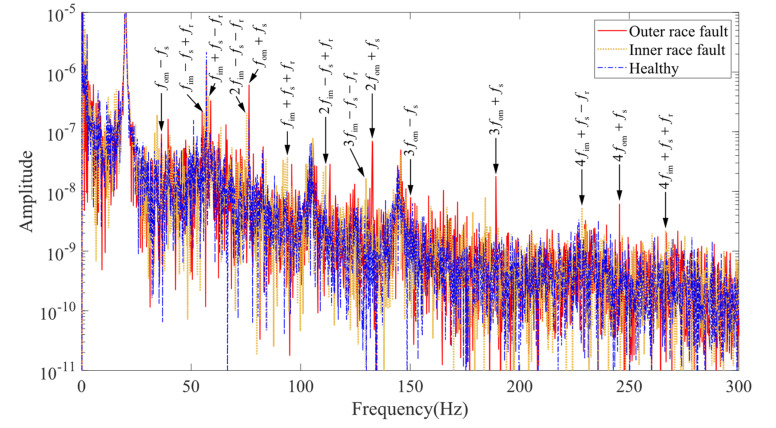
Stator current spectrum (simulation).

**Figure 18:**
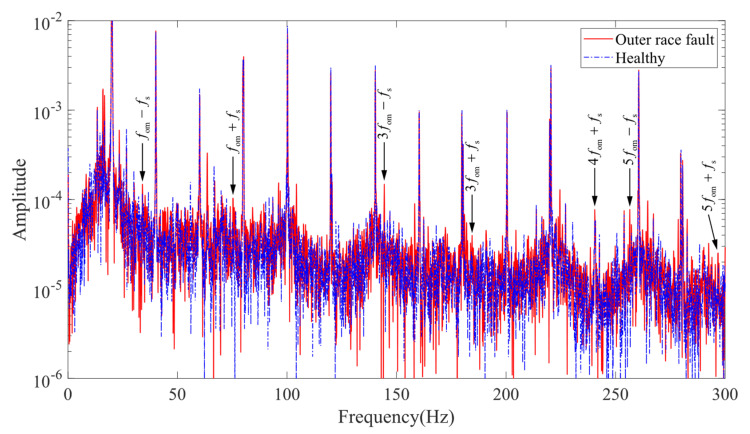
Stator current spectrum (experiment).

By checking the characteristic frequency peaks in the stator currents corresponding to the faults, the consistency between theoretical analysis, model development, and experiments can be verified. Taking the stator current signal recorded and saved at the same time with the vibration signal in the previous section, the spectrum of the simulated and experimental currents is plotted, as shown in Figure 17 and Figure 18.

In the spectrum of the simulated current signal shown in Figure 17, the prominent characteristic component located at the fault characteristic frequency can be checked. The characteristic frequencies with higher amplitude are $f_{\mathrm{im}}+f_{s}-f_{r}$,$2f_{\mathrm{im}}-f_{s}-f_{r}$,$2f_{\mathrm{im}}-f_{s}+f_{r}$,$f_{\mathrm{om}}+f_{s}$,$f_{\mathrm{im}}+f_{s}+f_{r}$,$2f_{\mathrm{om}}+f_{s}$,$3f_{\mathrm{om}}+f_{s}$,$4f_{\mathrm{im}}+f_{s}-f$,$f_{\mathrm{om}}+f_{s}$, etc., indicating that the air gap variation characteristics caused by the bearing faults can be reflected in the electromagnetic model part of the coupled model.

Figure 18 shows the experimentally measured stator current spectrum of the induction motor. The supply frequency (20 Hz) and its multiples are the main components of the spectrum. Affected by noise, only the characteristic frequencies $3f_{\mathrm{om}}-f_{s}$ and $5f_{\mathrm{om}}-f_{s}$ have obvious amplitude gain, and the characteristic frequencies $f_{\mathrm{om}}-f_{s}$, $f_{\mathrm{om}}+f_{s}$, $3f_{\mathrm{om}}+f_{s}$, $4f_{\mathrm{om}}+f_{s}$, and $5f_{\mathrm{om}}+f_{s}$ have weak amplitude gain. The comparison of simulation and experimental results verifies the correlation between the coupled model and the characteristic frequency formulation to a certain extent.

## 4. Conclusions

The simulation results show that the superposition of nonlinearities in the dynamical and electromagnetic models can induce complex dynamical behaviors reflected in the axial trajectories. These dynamical behaviors may be valuable for further analysis of the nonlinear and chaotic properties of the coupled model. Thus, although this paper is not a direct discussion of entropy and nonlinear mathematical problems, we still hope it can provide some reference for the extension of nonlinear theory in applications.

The construction of a model with effective coupling between dynamic model and electromagnetic model is beneficial for accurate analysis of the fault characteristics of the motor. Based on the velocity and air gap as the basic coupling paths, this study proposes an electromagnetic-dynamic coupled modeling method for induction motors considering UMP. In addition to the nonlinear factors such as bearing radial clearance and additional radial clearance, the rotor-bearing dynamics model based on the Hertzian contact theory introduces the effect of UMP caused by the radial displacement of the rotor. The change in air gap due to rotor vibration will affect the inductance calculation between the stator and rotor, thus serving as a path for the dynamics model to affect the electromagnetic model. The induction motor electromagnetic model based on the MCC theory feeds the rotor velocity directly into the dynamic model, while indirectly influencing the dynamic model through the UMP. In simulation results, the introduction of UMP makes the rotor show some special dynamical behaviors, and the amplitude and disorder of the vibration increases. In the vibration spectrum of the simulated signal, the UMP excites a speed modulation in normal and outer race fault conditions similar to that in the inner race fault case. The simulated spectrum curve located in the speed modulation range has a high amplitude, which is also reflected in the low frequency interval of the experimental signal spectrum. In the stator current simulation results of the coupled model, the frequency component associated with the fault can be found, which indicates the validity of the coupling path from the dynamic model to the electromagnetic model by air gap.

The coupled induction motor model integrating the rotor-bearing dynamic model and the MCC model has the ability to simulate a variety of mechanical and electrical faults, providing a more comprehensive approach for studying the dynamic behavior of motor rotors and fault characterization under different fault states. A correctly constructed coupled model is able to effectively simulate the motor state at different fault levels, which provides the possibility to track and predict the fault characteristics at each fault stage. Considering the industry’s demand for confidence in the prediction data, the coupled model constructed entirely based on the mechanism has the prerequisites to provide reliable results.

Due to the experimental conditions, this paper has been experimentally verified only for normal and outer race fault. In addition, because of the lack of measured data on the small rotor displacements, the roughness of the model validation work must be acknowledged. In further work, we will improve the experimental conditions and try to simulate the complex rotational motion of ball fault so as to incorporate the experiment of inner race fault and the simulation of ball fault into this study. In order to further verify the rotor dynamics in the simulation results, the measurement of the radial displacement of the rotor is also forthcoming.

## Figures and Tables

**Table 1:** Rotor-bearing dynamic model parameters.

Parameter	Value
bearing designation	SKF 6203
bearing outer race diameter	40 mm
bearing inner race diameter	17 mm
bearing ball diameter	6.747 mm
number of bearing balls	8
bearing clearance	3 × 10^−2^ mm
spalling width	3 mm
rotor mass	2.2299 kg
rotor damping	600 N∙s/m

**Table 2:** Multiple coupled circuit model parameters.

Parameter	Value
number of phases	3
number of poles	2
rated power	1/3 Hp
number of stator slots	24
number of stator coil turns	126
stator resistance	2.2 Ω
number of rotor bars	34
rotor length	60 mm
rotor bar resistance	8 × 10^−5^ Ω
end ring resistance	2.375 × 10^−5^ Ω
center radius of air gap	40.25 mm
initial length of air gap	0.7 mm

## Abbreviations

**Abbreviation**	**Meaning**
$\delta_{j}$	Contact deformation of the *j*-th ball
$x_{r}$	Rotor displacement in X direction
$y_{r}$	Rotor displacement in Y direction
$c_{l}$	Bearing radial clearance
$c_{lj}$	Additional radial clearance when the *j*-th ball passes through the spalling position
h	Equivalent spalling depth considering the ball does not touch the bottom of the spalling
$\theta_{\mathrm{spall}}$	Spalling starting angle position
$\Delta \theta_{\mathrm{spall}}$	Spalling angle
$w_{\mathrm{spall}}$	Spalling width
$d_{o}$	Bearing outer race diameter
$d_{i}$	Bearing inner race diameter
$d_{\mathrm{ball}}$	Bearing ball diameter
$K_{c}$	Contact stiffness
$\theta_{j}$	Angle position of the *j*-th ball
$m_{r}$	Rotor mass
$c_{r}$	Rotor damping
$F_{xi-\mathrm{restoring}}\left(i=1,2\right)$	Restoring force of the *j*-th bearing in the X direction
$F_{yj-\mathrm{restoring}}\left(j=1,2\right)$	Restoring force of the *j*-th bearing in the Y direction
$F_{x-\mathrm{magnetic}}$	Unbalanced magnetic pull of the rotor in the X direction
$F_{y-\mathrm{magnetic}}$	Unbalanced magnetic pull of the rotor in the Y direction
g	Gravitational acceleration
$D_{b}$	Bearing pitch diameter
$n_{\mathrm{ball}}$	Number of bearing balls
β	Bearing contact angle
$\lambda_{j}$	Contact coefficient of the *j*-th ball
$U_{s}$	Stator voltage vector
$I_{s,r}$	Stator and rotor current vectors
$R_{s,r}$	Stator and rotor resistance matrix
$\lambda_{s,r}$	Stator and rotor flux linkage vectors
$U_{\mathrm{sa},\mathrm{sb},\mathrm{sc}}$	Stator three-phase voltage
$i_{\mathrm{sa},\mathrm{sb},\mathrm{sc}}$	Stator three-phase winding currents
$R_{s}$	Single-phase stator winding resistance under the assumption of symmetrical stator
$\lambda_{\mathrm{sa},\mathrm{sb},\mathrm{sc}}$	Flux linkages on three-phase stator windings
$n_{\mathrm{bar}}$	Number of rotor bars
$R_{b}$	Single rotor bar resistance
$R_{e}$	End ring resistance
$i_{ri}\left(i=1,2,\ldots ,n_{b}\right)$	Current through the *i*-th rotor circuit
$\lambda_{ri}\left(i=1,2,\ldots ,n_{b}\right)$	Flux linkage through *i*-th rotor circuit
$i_{e}$	Current through the end ring
$\lambda_{e}$	Flux linkage through the end ring
$L_{\mathrm{ss}}$	Stator self-inductance matrix
$L_{\mathrm{rr}}$	Rotor self-inductance matrix
$L_{\mathrm{sr}}$	Stator–rotor mutual inductance matrix
$L_{\mathrm{rs}}$	Rotor–stator mutual inductance matrix
$L_{sisj}\left(i=1,2,3;j=1,2,3\right)$	Mutual inductance between stator phase *i* and stator phase *j*
$L_{sirj}\left(i=a,b,c;j=1,2,\ldots ,n_{b}\right)$	Mutual inductance between stator phase *i* and rotor circuit j
$L_{rirj}\left(i=1,2,\ldots ,n_{b},j=1,2,\ldots ,n_{b}\right)$	Mutual inductance between the *i*-th and *j*-th rotor circuits
$L_{b}$	Rotor bar leakage inductance
$L_{e}$	End ring leakage inductance
p	Number of motor poles
$\theta_{e}$	Electric angle
$T_{e}$	Electromagnetic torque
J	Rotor inertia
$\omega_{\mathrm{rm}}$	Rotor mechanical angular velocity
$T_{l}$	Load torque
$d_{\mathrm{ecc}}$	Rotor eccentricity
$\theta_{\mathrm{ecc}}$	Rotor eccentricity angle
e	Relative eccentricity
$g_{0}$	Uniformly distributed air gap length
g	Air gap length function
$\mu_{0}$	Permeability of air gap
r	Air gap left radius
l	Stack length
$n_{c_{1}}$	Turn function of coil $c_{1}$
$N_{c_{2}}$	Winding function of coil $c_{2}$
$g^{-1}$	Inverse function of air gap length
$\theta_{s}$	Stator space angle position
$\langle g^{-1}\rangle$	The average value of the air gap inverse function
t	Time
$\theta_{r}$	Rotor mechanical angular position
$\omega_{\mathrm{re}}$	Rotor electric angular velocity
$F_{s,r}$	Stator and rotor magnetomotive force amplitude
$A_{n}$	Fourier coefficient of air gap permeability
$n_{\mathrm{phase}}$	Number of phases/imputed number of phases
N	Total number of series turns per phase/imputed total number of series turns per phase
$k_{w}$	Fundamental winding factor
$k_{p}$	Fundamental wave pitch factor
$k_{d}$	Fundamental distribution factor
$f_{\mathrm{om}}$	Bearing outer race fault frequency (in vibration)
$f_{\mathrm{im}}$	Bearing inner race fault frequency (in vibration)
$f_{\mathrm{bm}}$	Bearing ball fault frequency (in vibration)
$f_{\mathrm{oe}}$	Bearing outer race fault frequency (in stator current)
$f_{\mathrm{ie}}$	Bearing inner race fault frequency (in stator current)
$f_{\mathrm{be}}$	Bearing ball fault frequency (in stator current)
$f_{\mathrm{cage}}$	Bearing cage rotation frequency
